# Elucidation of Potential Targets of San-Miao-San in the Treatment of Osteoarthritis Based on Network Pharmacology and Molecular Docking Analysis

**DOI:** 10.1155/2022/7663212

**Published:** 2022-01-18

**Authors:** Man Chu, Ting Gao, Xu Zhang, Wulin Kang, Yu Feng, Zhe Cai, Ping Wu

**Affiliations:** ^1^Faulty of Medical Technology, Shaanxi University of Chinese Medicine, Xianyang, China; ^2^The Affiliated Hospital of Shaanxi University of Chinese Medicine, Xianyang, China; ^3^Department of Pharmaceutics, General Hospital of Ningxia Medical University, Yinchuan, Ningxia, China; ^4^Department of Sports Medicine and Arthroscopy, General Hospital of Ningxia Medical University, Yinchuan, Ningxia, China; ^5^Department of Allergy, Immunology and Rheumatology, Guangzhou Women and Children's Medical Center, Guangzhou, China; ^6^Institute of Chinese Medicine and State Key Laboratory of Research on Bioactivities and Clinical Applications of Medicinal Plants, The Chinese University of Hong Kong, Hong Kong

## Abstract

**Background:**

To examine the potential therapeutic targets of Chinese medicine formula San-Miao-San (SMS) in the treatment of osteoarthritis (OA), we analyzed the active compounds of SMS and key targets of OA and investigated the interacting pathways using network pharmacological approaches and molecular docking analysis.

**Methods:**

The active compounds of SMS and OA-related targets were searched and screened by TCMSP, DrugBank, Genecards, OMIM, DisGeNet, TTD, and PharmGKB databases. Venn analysis and PPI were performed for evaluating the interaction of the targets. The topological analysis and molecular docking were used to confirm the subnetworks and binding affinity between active compounds and key targets, respectively. The GO and KEGG functional enrichment analysis for all targets of each subnetwork were conducted.

**Results:**

A total of 57 active compounds and 203 targets of SMS were identified by the TCMSP and DrugBank database, while 1791 OA-related targets were collected from the Genecards, OMIM, DisGeNet, TTD, and PharmGKB databases. By Venn analysis, 108 intersection targets between SMS targets and OA targets were obtained. Most of these intersecting targets involve quercetin, kaempferol, and wogonin. Moreover, intersecting targets identified by PPI analysis were introduced into Cytoscape plug-in CytoNCA for topological analysis. Hence, nine key targets of SMS for OA treatment were obtained. Furthermore, the potential binding conformations between active compounds and key targets were found through molecular docking analysis. According to the DAVID enrichment analysis, the main biological processes of SMS in the treatment of OA include oxidative stress, response to reactive oxygen species, and apoptotic signaling pathways. Finally, we found wogonin, the key compound in SMS, might play a pivotal role on Toll-like receptor, IL-17, TNF, osteoclast differentiation, and apoptosis signaling pathways through interacting with four key targets.

**Conclusions:**

Therefore, this study elucidated the potential active compounds and key targets of SMS in the treatment of OA based on network pharmacology.

## 1. Introduction

Osteoarthritis (OA) is a common degenerative joint disease worldwide, especially affecting the health and quality of life of patients at the middle-age or older. OA is a heterogeneous disease with multiple phenotypes and triggers, such as age, cartilage damage, metabolic disorders, subchondral bone remodeling, and inflammation [1]. Obesity, sex, age, genetics, diet, traumatic injury, abnormal joint load, and joint alignment are the risk factors for OA [2]. OA is mainly characterized by a low level of intra-articular inflammation and the degeneration or destruction of articular cartilage. These pathological features usually lead to pain of knee joint, a significant decline in patients' quality of life, sleeping disorders, and even a series of mental and psychological disorders [3–5].

At present, there are two main strategies in the treatment of OA. The first strategy is based on the western medicine theory, which focuses on eliminating osteophytes, promoting articular cartilage repair, and correcting imbalanced mechanical burden of joint. Western medicine currently used, such as nonsteroid anti-inflammatory drugs or selective COX-2 inhibitors, mainly aim at alleviating joint pain. However, they have little effect on the recovery of chondrogenic development and the improvement of OA progression. Besides, the side effects of western medicine limit their long-term application [6–8]. Biological inhibitors such as tumor necrosis factor (TNF) and interleukin- (IL-) 6 inhibitors can relieve the inflammatory symptoms in rheumatoid arthritis, but their efficacy is limited in OA [9]. The second strategy is based on the traditional Chinese medicine (TCM). OA can be treated through various approaches such as oral administration of herbal medicine, topical treatment, physiotherapy, and so on. TCM formula San-Miao-San (SMS) derived from the Chinese Medical History “Orthodox Medicine Science-Numbness” is a novel formula for treating OA. SMS has been used to treat arthralgia syndrome for hundreds of years. It is composed of Rhizoma Atractylodis/Cang Zhu (CZ), Cortex Phellodendri/Huang Bo (HB), and Achyranthis Bidentatae/Niu Xi (NX) [10]. HB with bitter nature is good at clearing dryness, dampness, and internal heat. CZ powder with bitter nature is good at invigorating spleen and clearing dryness and dampness. NX tonifies liver and kidney and strengthens muscles and bones. It is specially used to treat numb syndromes and weakness of feet [11]. Several studies have shown that SMS or modified SMS improves the efficacy of western medicine for treating rheumatoid arthritis and gouty arthritis [10, 12, 13]. Our previous study demonstrated that *Ganoderma lucidum*-SMS combined with hyaluronic acid gel can alleviate cartilage degeneration in traumatic OA [14].

Treatment using TCM often involves the synergistic action of multicomponents and multitargets. The principle theory of TCM for treating chronic disease is consistent with the basic theory of network pharmacology. Therefore, this study intends to use the approaches of network pharmacology and molecular docking to discover the mechanism of SMS for OA treatment, so as to provide a theoretical support for further investigation.

## 2. Materials and Methods

### 2.1. Screening Active Compounds and Targets of Compounds in SMS

Traditional Chinese Medicine Systems Pharmacology Database (Version:2.3, https://old.tcmsp-e.com/tcmsp.php) were used to search Chinese medicine composition of SMS with keywords “Cortex Phellodendri,” “Radix Achyranthis Bidentatae,” and “Rhizoma Atractylodis” [15]. Oral bioavailability (OB) and drug-likeness (DL) were used to screen each compound, and those compounds that met OB ≥ 30% and DL ≥ 0.18 were considered to be the potential active components. The active targets of components in SMS were screened out from the DrugBank database (https://www.drugbank.ca/) [16].

### 2.2. Screening OA-Related Disease Targets in Human

The five databases including Genecards (https://www.genecards.org/) [17], OMIM (https://omim.org/) [18], DisGeNet (https://www.disgenet.org/) [19], TTD (https://db.idrblab.net/ttd/) [20], and PharmGKB (https://www.pharmgkb.org/) [21] were screened to collect OA-related disease targets by searching the keyword “knee osteoarthritis (UMLS CUI: C0409959) or osteoarthritis.”

### 2.3. Compound-Target Network Construction

The compound-target network of SMS was constructed by using Cytoscape software (version 3.7.2, Boston, MA, USA) [22]. After sorting and classifying the active components of SMS and its corresponding targets, the effective compounds of SMS and common target genes of compounds and OA were used to create network nodes. The relationship between the nodes was illustrated by connecting lines in the Cytoscape software. Therefore, a network between the active compounds of SMS and the target genes of OA could be established. After that, the compound-target network was exported with the degree centrality of targets.

### 2.4. Construction of Protein-Protein Interaction (PPI) Network

The PPI network of target was constructed through the Search Tool for the Retrieval of Interacting Genes/Proteins (STRING, https://string-db.org/) online database [23]. “Multiple proteins names” were selected and the screened genes were input. “*Homo sapiens*” was selected as the target species, and the PPI network with the minimum required interaction score ≥0.4 was constructed, without isolated proteins.

### 2.5. Topological Optimization of Interaction Network

The key targets of interaction network were analyzed in the topological analysis. The CytoNCA plug-in tool of Cytoscape was used to analyze the topological parameters of all nodes in PPI network in the way of being without weight, including degree centrality (DC), betweenness centrality (BC), closeness centrality (CC), eigenvector centrality (EC), information centrality (IC), and local average connectivity-based method (LAC). The median values of the topological parameters were considered as a standard rule to filter targets of compounds. The obtained nodes were the key nodes in the interaction network.

### 2.6. Bioinformatics Analysis

David Bioinformatics Resources 6.7 (https://david-d.ncifcrf.gov/) was used for gene ontology (GO) and Kyoto Encyclopedia of Genes and Genomes (KEGG) enrichment analysis [24]. In this study, the Benjamini–Hochberg method was used to control the false discovery rate (FDR) of multiple hypothesis testing and corrected *p* < 0.05 was significant [25]. The analysis of GO functional enrichment and KEGG pathway enrichment was presented in the visual graphics and data tables.

### 2.7. Molecular Docking Analysis

AutoDock software (AutoDock 4.2, San Carlos, CA, USA) was used for protein-compound docking analysis [26]. In brief, the two-dimensional (2D) structure of the compound was obtained from the NCBI PubChem structure file, and the three-dimensional (3D) structure was constructed by ChemBio 3D Ultra software (Cambridgesoft, version 14.0) after energy minimization. The crystal structure of receptor protein was obtained from the PDB website (https://www.rcsb.org/) [27]. The crystal structure of ligand/compound was extracted by PyMoL software (San Carlos, CA, USA). Use AutoDock software to add polar hydrogen to the entire receptor, and the grid box was set to contain the entire receptor region. In all docking studies, 20 docking conformations were generated for each pair of ligand and receptor. The affinity energy was calculated using genetic algorithm. A binding energy less than 0 indicated that the ligand could spontaneously bind to the receptor. It was generally believed that if the ligand binds to the receptor stably, the lower the energy fraction, the greater the binding possibility [26].

## 3. Results

### 3.1. The Active Compounds and Potential Targets of Compounds in SMS

A total of 365 components were found through searching the Traditional Chinese Medicine Systems Pharmacology (TCMSP) database, including 49 components from Rhizoma Atractylodis/CZ, 176 components from Radix Achyranthis Bidentatae/NX, and 140 components from Cortex Phellodendri/HB. The active ingredients in SMS were screened according to the principle of pharmacokinetics. If their OB ≥ 30% and DL ≥ 0.18 [15], they were considered as the active compounds. A total of 57 potential active compounds were obtained after eliminating the duplication (Table 1), and a total of 203 targets corresponding with these active compounds were confirmed by searching the DrugBank database (Supplementary Table S1). After screening, there were 9, 20, and 37 active compounds in CZ, NX, and HB, respectively.

### 3.2. Compound-Target Network

**Figure 1:**
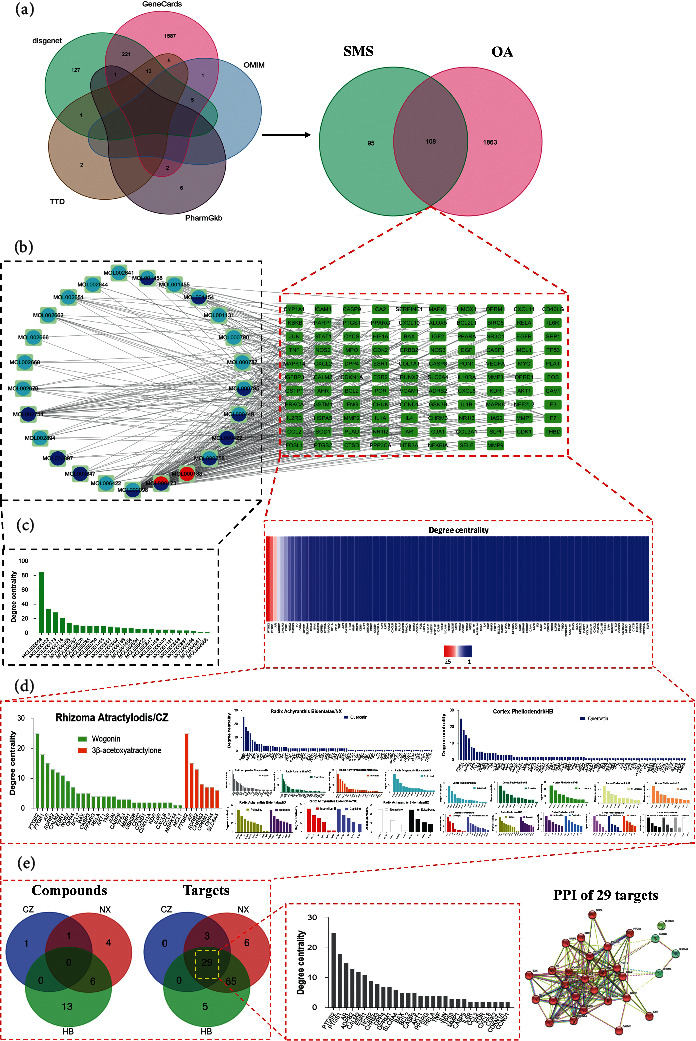
Analysis of active compound-target network of SMS for OA treatment. (a) Venn analysis of 5 databases for OA related disease targets, and intersection targets between OA-related targets and targets of SMS active compounds. (b) Analysis of active compound-target network of SMS. Red circle: compounds from Rhizoma Atractylodis. Blue circle: compounds from Radix Achyranthis Bidentatae. Indigo circle: compounds from Cortex Phellodendri. (c) The degree centrality of SMS active compounds and intersection targets. The reddish color indicates smaller degree centrality and vice versa. (d) The degree centrality of intersection targets of active compounds among CZ, HB, and NX. The bar charts with different colors represent the different active compounds corresponding intersection targets. (e) Venn diagram shows the intersection compounds and targets among CZ, HB, and NX. The bar chart shows the degree centrality of 29 intersection targets among CZ, HB, and NX. The 29 intersection targets show 3 clusters in protein-protein interaction network.

Although we have found the potential active compounds and their corresponding targets in SMS, the therapeutic mechanism of SMS in OA has not been fully understood. In order to understand more intuitively how the potential active compounds of SMS play a therapeutic role on OA, a total of 2239 OA-related disease targets were obtained. They include 368 targets from DisGeNet database, 1835 targets from GeneCards database, 6 targets from Online Mendelian Inheritance in Man (OMIM) database, 9 targets from Pharmacogenetics Knowledge Base (PharmGKB) database, and 21 targets from Therapeutic Target Database (TTD) (Figure 1(a), Supplementary Table S2). After eliminating the duplicates by Venn analysis, we found that 1971 targets were related to OA disease, and 108 intersection targets between OA-related targets and targets of SMS active compounds (Figure 1(a)). These intersection targets were considered as the active targets of SMS in the treatment of OA (Figures 1(a) and 1(b)). Therefore, a component-target network containing 25 active compounds of SMS and 108 intersection targets was constructed using Cytoscape software (Figure 1(b)). After further analysis of the component-target network, the degree centrality of the active target was determined by its connection. Through the analysis of the degree centrality of 25 SMS active compounds and 108 intersection targets in each herb (Figure 1(c)), SMS active compounds with the highest degree centrality were quercetin (MOL000098, degree centrality = 85), kaempferol (MOL000422, degree centrality = 34), and wogonin (MOL000173, degree centrality = 29). Quercetin derived from NX and HB, kaempferol from NX, and wogonin (degree centrality = 29) from CZ and NX were flavonoid compounds and had the largest number of active targets (Figure 1(c)): quercetin (85 targets), kaempferol (34 targets), and wogonin (29 targets). Meanwhile, we found that the top four targets with the highest degree centrality among the 108 intersection targets were prostaglandin-endoperoxide synthase- (PTGS-) 2 (degree centrality = 25), PTGS1 (degree centrality = 18), androgen receptor (AR, degree centrality = 15), and beta-2 adrenergic receptor gene (ADRB2, degree centrality = 13) (Figure 1(c)). Two of them were coding enzymes (PTGS2 and PTGS1), and the other two were receptors (AR and ADRB2). Moreover, all of them could be found in the diagrams of degree centrality of quercetin, kaempferol, and wogonin (Figure 1(d)), and in the diagrams of degree centrality of 29 intersection targets, which were extracted by Venn analysis among CZ, HB, and NX (Figure 1(e)). Then, three clusters of these 29 intersection targets were formed by PPI network.

### 3.3. Analysis of Key Targets of SMS in the Treatment of OA

**Figure 2:**
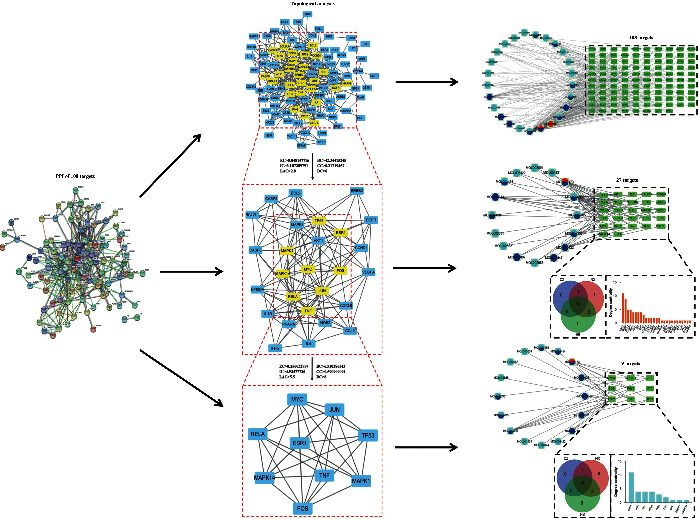
Topological analysis of intersection targets between OA-related targets and targets of SMS active compounds. The 108 intersection targets shown in protein-protein interaction network are performed for topology network analysis twice. The parameters such as BC, CC, DC, EC, IC, and LAC represent the filter conditions. The yellow rectangles represent the key targets in the subnetwork obtained from topology analysis. The active compounds-key targets network of each screening analysis is presented with the degree centrality of key targets and the intersection targets among CZ, HB, and NX.

As we know, the physiological functions of proteins were usually regulated by protein interactions and their corresponding pathways. In order to uncover the functions and mechanism of SMS in the treatment of OA, a PPI network of the 108 intersection targets identified above was constructed by online String v11.0 (Figure 2). The key targets of SMS could be further identified by the topological analysis of the PPI network (Figure 2). As the median values of each intersection target in DC, BC, CC, EC, IC, and LAC were 42.344, 0.217, 6.000, 0.048, 3.107, and 2.800, respectively, the nodes that were greater than these median values were filtered. A total of 27 nodes that met the requirements were therefore selected. All of them were used to construct an interactive sub-network for further topological analysis (Figure 2). Through similar screening criteria, the median values of BC, CC, DC, EC, IC, and LAC were calculated, 9 refined key targets (estrogen receptor- (ESR-) 1, protooncogene c-Fos (FOS), mitogen-activated protein kinase- (MAPK-) 1, MAPK14, Rela (p65 NF-*κ*B), TP53, TNF, transcription factor activator protein-1 (Jun), and Myc proto-oncogene protein (Myc)) were found because their median values of BC, CC, DC, EC, IC, and LAC were greater than 6.510, 0.591, 8.000, 0.156, 4.935, and 5.500, respectively (Figure 2). As the same compounds of SMS could interact with both 27 core nodes and 9 key targets in the compound-target network, they may play a key role in OA treatment. We then combined the degree centrality of targets and analyzed the key targets of active compounds in each herb. By Venn analysis of these targets among the three herbs (CZ, HB, and NX), it was found that 12 targets could correspond with the active compounds of all herbs, and 5 of them were the key targets (Figure 2). We also found that three compounds (quercetin, kaempferol, and wogonin) could interact with these key targets (Supplementary Tables S3–S5), which also implied that these three compounds might be the key active compounds of SMS in OA treatment.

### 3.4. Bioinformatics Analysis

**Figure 3:**
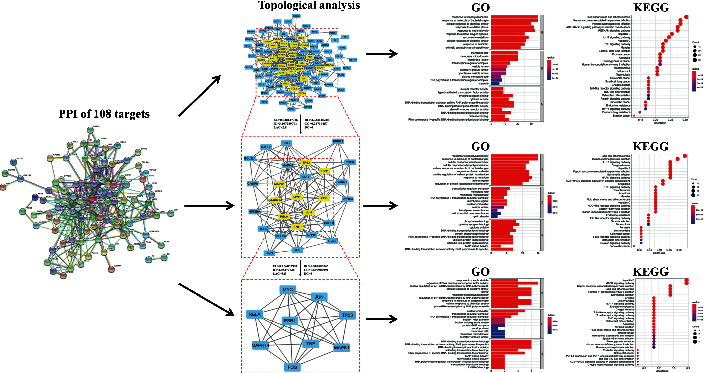
GO function enrichment and KEGG pathway enrichment analysis of PPI network. The important biological processes (BP), cell composition (CC), and molecular function (MF) of GO function enrichment analysis of intersection targets between OA-related targets and targets of SMS active compounds are followed by each topological analysis. The results of the GO function enrichment analysis present in bar chart. The length of the bar in the figure indicates the number of targets participating in each function. The results of the KEGG pathway enrichment analysis are plotted. The size of the circle in the picture indicates the number of targets participating in the path. The reddish color indicates smaller *p* value and vice versa.

To have a more macroscopic and overall understanding of the physiological processes and biological function of the intersection targets, we performed the GO functional enrichment analysis and KEGG pathway enrichment analysis on all targets of each subnetwork. The most important biological processes (BP), cell compositions (CC), and molecular functions (MF) involved in these targets were shown in Figure 3 (corrected *p* < 0.05). They include 2 common BP (response to lipopolysaccharide, and response to molecule of bacterial origin), 4 common CC (membrane raft, membrane microdomain, transcription regulator complex, and RNA polymerase II transcription regulator complex), and 3 common MF (DNA-binding transcription factor binding; DNA-binding transcription activator activity, RNA polymerase II-specific; and RNA polymerase II-specific DNA-binding transcription factor binding). Through KEGG analysis, the most important signaling pathways were shown in Figure 3 (corrected *p* < 0.05), including 15 common KEGG pathways, such as Toll-like receptor (TLR), IL-17, and TNF signaling pathways, osteoclast differentiation, and apoptosis. The comprehensive results of GO and KEGG enrichment analysis were listed in Supplementary Tables S6–S11.

### 3.5. Comprehensive Analysis of Compound-Target Network with Molecular Docking

**Figure 4:**
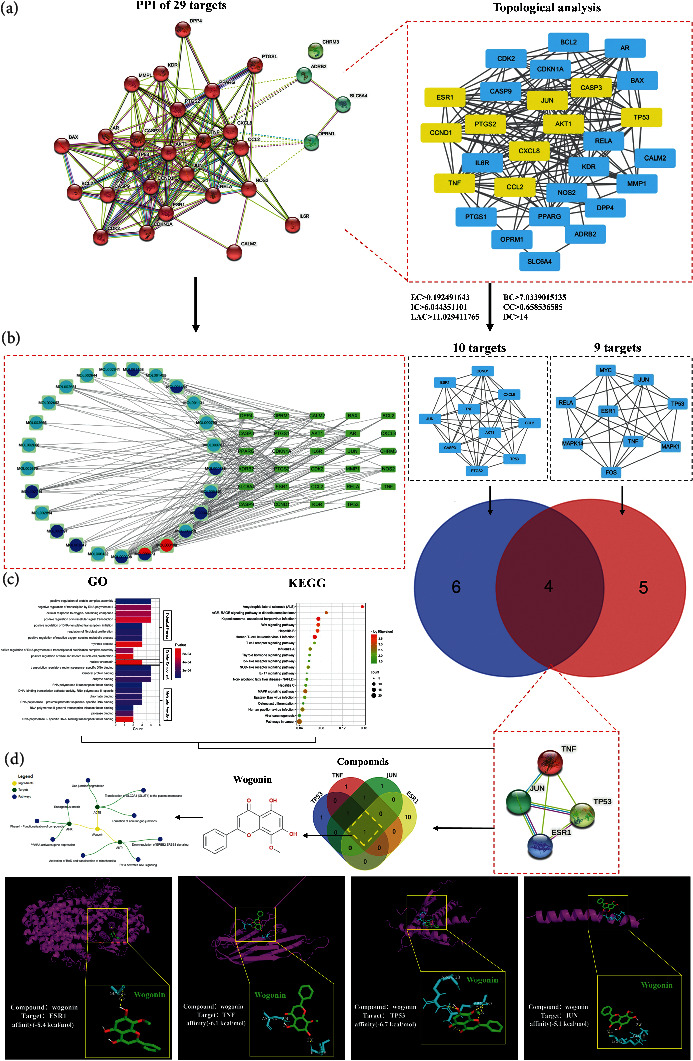
Comprehensive analysis of SMS active compounds-key targets network with molecular docking. (a) The protein-protein interaction network and topological analysis of 29 intersection targets. There are 10 yellow rectangles obtained from topology analysis that represent the key targets, compared with previous found 9 key targets for Venn analysis. (b) The interactive network of SMS active compounds and 29 intersection targets. The interaction of 10 and 9 key targets present in each subnetwork. (c) The KEGG pathway enrichment and GO function enrichment analysis of 4 intersection targets in PPI network. These intersection targets are derived from Venn analysis of subnetworks. The reddish color indicates smaller *p* value and vice versa. (d) The molecular structure presents the active compound wogonin, which is derived from Venn analysis of above 4 key targets' corresponding compounds. The targets and pathways of wogonin are present in central network diagram, which is output from a TCM database (Integrative Pharmacology-Based Research Platform of Traditional Chinese Medicine, TCMIP v2.0). Then, the three-dimensional conformations of wogonin and corresponding targets including the affinity energy present in the molecular docking diagram. The yellow rectangles present the spatial docking of wogonin in each target.

To have an in-depth understanding of the interactions between active compounds of SMS and their targets, a comprehensive analysis of molecular docking between the active compounds and targets was performed to explore and verify the potential key compounds of SMS in OA treatment (Figure 4). Therefore, the PPI network was further analyzed based on the compound-target network, which was composed of 25 active compounds of SMS and 29 intersection targets (Figure 4(b)). In addition, the topological analysis for the 29 intersection targets found above was performed, and 10 potential key targets (AKT1, CASP3, CCL2, CCND1, CXCL8, ESR1, JUN, PTGS2, TNF, and TP53) were obtained through previous screening criteria. Their median values of BC, CC, DC, EC, IC, and LAC were greater than 7.034, 0.659, 14.000, 0.192, 6.044, and 11.029, respectively (Figure 4(a)). When compared with the previous 9 key targets obtained from the Venn analysis, we found 4 common targets (ESR1, TNF, TP53, and JUN) which could form a PPI network. To further understand the biological function of these targets in OA, we carried out GO function enrichment and KEGG pathway enrichment analysis (Figure 4(c)). From the GO enrichment analysis, most of these targets were enriched for negative regulation on the transcription by RNA polymerase II (GO: 0000122), nuclear chromatin (GO: 0000790), and RNA polymerase II transcription factor binding (GO: 0001085), implicating that they might be the main functions of these targets. From the KEGG enrichment analysis, we found that the main pathways involved were MAPK signaling pathway (hsa04010) and Wnt signaling pathway (hsa04310), as the number of genes involved in these pathways was high. It may imply that these signaling are the main pathway involved in these targets (Figure 4(c) and Supplementary Tables S12 and S13). Therefore, the common active compounds of these targets were obtained by Venn analysis. We found wogonin was the only compound that formed a central network with its corresponding targets and pathways. The binding affinity between wogonin and 4 common targets were evaluated by molecular docking (Figure 4(d)). The results of the minimum energy fraction from 20 docking schemes of each group were selected as the docking structure model. The lower energy fraction (less than −5 kcal/mol) indicated the better degree of a ligand binding to its receptor in the molecular docking analysis [26]. The identification symbols of each target protein in the Protein Data Bank (PDB) were shown as follows: ESR1 (3OS8), TP53 (1Sal), TNF (7Kp9), and JUN (5Fv8) [27]. Among them, most of the binding between wogonin and targets was through the formation of hydrogen bonds or pi bonds, and some interactions were produced by van der Waals force. As the docking score of ESR1 (−8.4 kcal/mol) was the highest, the combination of ESR1 and wogonin may play an important role in targeting the key process of OA.

## 4. Discussion

The syndrome of knee arthralgia described in the “Su Wen⋯Chang Ci Jie Lun” of TCM literature belongs to the category of OA. According to the theory of TCM, OA is the functional deficiency of liver and kidney, which involves vessel blocking and joint pain. Therefore, it is believed that the multidrugs and multitargets approach should be considered in the treatment of OA [28]. Different from the “one monomer compound for one disease treatment” methodology, network pharmacology can be used to study the potential mechanisms of various components of TCM formulas like SMS in OA treatment. In the previous study, we have found that *Ganoderma lucidum*-SMS can directly alleviate cartilage degeneration in the knee joint by activating the chondrogenic signaling pathway in a traumatic OA rat model [14]. Additionally, *Ganoderma lucidum*-SMS plays an indirect regulatory role on the integrity of subchondral bone microstructure that offers a stabilized physiological environment for the recovery of the damaged cartilage. Meanwhile, besides the synergistic anti-inflammatory effect of *Ganoderma lucidum* and SMS, a target-specific signaling pathway (MAPK pathway) has been found during OA progression. However, the active ingredients of *Ganoderma lucidum*-SMS and their targets in OA, as well as the interactive relationship and key genes of the signaling pathway involved, are not well illustrated. Therefore, through the approaches of network pharmacology and molecular docking, we discovered the potential active ingredients of SMS and the related targets to provide a potential mechanism of SMS in OA treatment.

In this study, the potential active compounds and targets of SMS and OA-related target proteins were obtained from TCMSP, DrugBank, GeneCards, OMIM, DisgenET, and PharmGKB databases. The main targets of SMS on OA were obtained by Venn analysis, and the sources of the intersection targets were further analyzed. Quercetin, kaempferol, and wogonin were found to be the key active components of SMS in the treatment of OA. Quercetin, a polyphenolic flavonoid, has been reported to possess anti-inflammatory, antioxidant, and antiallergic activities. It is reported that intra-articular injection of quercetin may inhibit the production of IL-1*β* and TNF-*α* by regulating TLR-4/NF-*κ*B pathway and reduce the degradation of cartilage and the apoptosis of chondrocytes in an OA rat model [29]. It is confirmed that quercetin can significantly inhibit the expression of matrix metalloproteinases and inflammatory mediators and can promote the production of cartilage synthesis factors in chondrocytes [29]. In addition, quercetin plays an antiapoptotic role in chondrocytes by reducing intracellular reactive oxygen species (ROS), restoring mitochondrial membrane potential, and inhibiting the caspase 3 pathway [30]. Other studies have shown that quercetin treatment can reduce oxidative stress, endoplasmic reticulum stress, and chondrocytes apoptosis [31]. Kaempferol is also a flavonoid compound and has antiapoptotic and anti-inflammatory pharmacological activities. It has been reported that kaempferol inhibits the production and release of inflammatory cytokines (TNF-*α* and IL-6), mediators (nitric oxide/NO and prostaglandin E2), and signaling molecules (SRC, SYK, and IRAK4), as well as the release of ROS [32]. It was found that kaempferol significantly reduced the production of proinflammatory mediators by inhibiting the NF-*κ*B pathway, which suggests a prominent anti-inflammatory and antiarthritis effects of kaempferol in IL-1*β*-stimulated rat chondrocytes [33]. Wogonin is also a flavonoid that exhibits anti-inflammatory and antioxidant effects on chondrocytes and cartilage explants by activating ROS/extracellular regulated protein kinases (ERK)/nuclear factor erythroid 2-related factor 2 (NRF2) signaling pathway. These signaling pathways were involved in oxidative stress, inflammatory response, and matrix degradation in OA [34]. Matrix metalloproteinase- (MMP-) 3 plays an important role in the pathophysiology of OA through the degradation of extracellular matrix components like proteoglycan. One study reported that wogonin could downregulate the expression of MMP-3 in chondrocytes to protect cartilage degradation [35, 36]. In summary, all of the above key active components of SMS may play a synergistical role on OA-related targets to perform the antiapoptotic, antioxidant, and anti-inflammatory effects.

In this study, the key targets of SMS for the treatment of OA obtained by Venn analysis, PPI network, topological analysis, and molecular docking analysis include ESR1, FOS, MAPK1, MAPK14, Rela, TP53, TNF, Jun, and Myc. The association between the ESR gene polymorphisms and OA has been found in several studies [37, 38]. For example, ESR variants, which are the genetic markers of OA, are associated with the susceptibility of joint pain in women [38]. What we found is wogonin can bind to the glycine (Gly521) residue in the ligand-binding domain of ESR1 (shown in Figure 4(d)). In addition, the increased c-Jun protein in IL-1*β*-stimulated chondrocytes can form a heterodimer of activator protein-1 (AP-1) with FOS [39]. Meanwhile, the IL-1*β*-activated c-Jun N-terminal kinase (JNK), one of the important branches of MAPK family, can form a vital JNK-c-Jun pathway for chondrocyte apoptosis [39]. Thus, JNK and c-Jun are often indicated to be the important targets of anti-inflammatory therapy in chondrocyte apoptosis [40]. Therefore, the progression of OA can be effectively alleviated by regulating the JNK signaling pathway [41]. The mechanism of the MAPK signaling pathway in OA has been extensively studied. JNK and p38 MAPK signaling pathways are related to the apoptosis of chondrocytes in OA. The osteoblasts derived from subchondral bone may inhibit the anabolism and promote catabolism of chondrocytes in OA through the ERK1/2 signaling pathway [42, 43]. Hence, we believe that MAPK, as a mediator to regulate the expression of proinflammatory cytokines and the downstream MMPs, may be involved in the potential pathway for preventing OA progression [44]. Besides MAPK, the level of phosphorylated Rela is also significantly increased in OA. It also upregulates the expression of proinflammatory cytokines, chemokines, and matrix degrading enzymes [45]. In this study, several cell compositions like the transcription regulator complex and molecular function like DNA-binding transcription activator activity were identified (shown in Figure 3). Because the phosphorylated p53 accumulated induce chondrocytes apoptosis or senescence under the stimulation of nitric oxide (NO), IL-1*β,* and mechanical stress in OA, we believe TP53 may involve in the above cell compositions and molecular functions [46, 47]. Most importantly, we found the 4 major targets of wogonin (shown in Figure 4(d)). TNF-*α* is one of them, and it is considered to be an important factor in cartilage degeneration during the development of OA. The inhibitor of TNF-*α* also has an inhibitory effect on the progression of OA [48]. Interestingly, in this study, we found that wogonin could interact with TNF-*α* (7Kp9) through forming hydrogen bonds with its amino acid residues asparagine (Asn40) and isoleucine (Ile136). Meanwhile, we predicted the potential interactive sites and affinity energies between wogonin and amino acid residues of each target. Most of the potential bindings between wogonin and targets are through the hydrogen bonds (shown in Figure 4(d)). However, the real effect and mechanism of interaction between SMS active compounds and OA-related key targets require further experimental verifications.

Besides the key targets found in SMS-treated OA and their interactive network, the mechanism of the active compounds participated in the cellular function and signaling pathways is clarified in GO and KEGG enrichment analysis (shown in Figure 3). GO functional analysis of genes shows that these target genes play a potential role on a series of biological processes by affecting apoptotic signaling pathways, cellular responses to oxidative stress, responses to nutrient levels, ROS, and metal ions. Many studies have shown that the imbalanced coupling mechanism between chondrocyte proliferation and apoptosis are two of the reasons for OA progression [49, 50]. For instance, chondrocyte apoptosis is the main cause for OA progression, and excessive oxidative stress triggers the activation of apoptosis- or necrosis-related pathways in chondrocytes, thereby leading to cartilage degeneration in OA [51]. Oxidative stress can also promote the development of inflammation [52]. For example, ROS can directly or indirectly reduce the cartilage matrix by activating all of the potential MMPs to increase proinflammatory cytokines and chemokines, thereby resulting in cell apoptosis [53, 54].

From the results of KEGG enrichment analysis (shown in Figure 3), the main signaling pathways involved in SMS treated OA are TLR, IL-17, and TNF signaling pathways, osteoclast differentiation, and apoptosis. The differentiated osteoclasts from synovial membrane involve bone resorption in the progressive OA [55–57]. Nuclear factor- (NF-) ĸB ligand (RANKL), a member of the TNF cytokine ligand superfamily, is essential for osteoclast differentiation and can also regulate bone resorption and prevent osteoclasts from death [58]. An upregulation of RANKL in chondrocytes is correlated with the cartilage degeneration and osteoclast density in the mineralized tissues [59]. It is implied that RANKL could be a key signaling molecule in the crosstalk between the cartilage and bone. The relationship between the cartilage RANKL score and osteoclast density strongly suggests that the chondrocytes could modulate osteoclast recruitment for subchondral bone remodeling in OA [60]. Moreover, the interaction of RANKL and RANK induces osteoclast progenitor to differentiate into osteoclast. Subsequently, the RANK-RANKL complex induces an activation of TNF receptor-associated factor 6 (TRAF6), which in turn involves the activation of NF-ĸB and MAPKs, including p38 and JNK [61]. In addition, TLR plays an important role in nonspecific immunity and acts as a bridge between specific immunity and nonspecific immunity. The signaling pathway involved in this receptor is closely related to immune or inflammatory diseases. Therefore, the activation of TLR4 can trigger the downstream NF-*κ*B signaling and upregulate the expression of IL-1*β* and TNF-*α* to exacerbate inflammation in OA [62, 63].

## 5. Conclusions

The existing treatment of OA still cannot meet the current clinical demands, and the side effects of long-term use of western medicine should not be ignored. TCM has the “multicomponents and multitargets” characteristics and has been used to treat various chronic diseases in a systematic and holistic manner. This study explored the mechanism of SMS in the treatment of OA based on network pharmacology. We have found that wogonin is the key compound in SMS, which may play a therapeutic role through four key targets (ESR1, JUN, TP53, and TNF) and act on TLR, IL-17, and TNF, osteoclast differentiation, and apoptosis signaling pathways. The results of network pharmacological analysis provide a valuable reference for elucidating the anti-inflammatory mechanism of SMS and its compounds in the treatment of OA and discovering new drugs from TCM. However, the corresponding targets of specific active components of SMS and the synergistic effect between them require further study.

## Figures and Tables

**Table 1:** The potential active compounds in SMS.

	MOL ID	MOL Name	OB (%)	DL	CM
1	MOL000422	Kaempferol	41.88	0.24	NX
2	MOL001006	Poriferasta-7, 22E-dien-3*β*-ol	42.98	0.76	NX
3	MOL002714	Baicalein	33.52	0.21	NX
4	MOL002776	Baicalin	40.12	0.75	NX
5	MOL002897	Epiberberine	43.09	0.78	NX
6	MOL003847	Inophyllum E	38.81	0.85	NX
7	MOL004355	Spinasterol	42.98	0.76	NX
8	MOL012461	28-Norolean-17-en-3-ol	35.93	0.78	NX
9	MOL012505	Bidentatoside, ii_qt	31.76	0.59	NX
10	MOL012537	Spinoside A	41.75	0.4	NX
11	MOL012542	*β*-Ecdysterone	44.23	0.82	NX
12	MOL000622	Magnograndiolide	63.71	0.19	HB
13	MOL000762	Palmidin A	35.36	0.65	HB
14	MOL000787	Fumarine	59.26	0.83	HB
15	MOL000790	Isocorypalmine	35.77	0.59	HB
16	MOL001131	Phellamurin_qt	56.6	0.39	HB
17	MOL001455	(S)-canadine	53.83	0.77	HB
18	MOL001771	Poriferast-5-en-3beta-ol	36.91	0.75	HB
19	MOL002636	Kihadalactone A	34.21	0.82	HB
20	MOL002641	Phellavin_qt	35.86	0.44	HB
21	MOL002644	Phellopterin	40.19	0.28	HB
22	MOL002651	Dehydrotanshin one II A	43.76	0.4	HB
23	MOL002652	Delta7-dehydrosophoramine	54.45	0.25	HB
24	MOL002656	Dihydroniloticin	36.43	0.81	HB
25	MOL002659	Kihadanin A	31.6	0.7	HB
26	MOL002660	Niloticin	41.41	0.82	HB
27	MOL002662	Rutae carpine	40.3	0.6	HB
28	MOL002663	Skimmianin	40.14	0.2	HB
29	MOL002666	Chelerythrine	34.18	0.78	HB
30	MOL002668	Worenine	45.83	0.87	HB
31	MOL002670	Cavidine	35.64	0.81	HB
32	MOL002671	Candletoxin A	31.81	0.69	HB
33	MOL002672	Hericenone H	39	0.63	HB
34	MOL002673	Hispidone	36.18	0.83	HB
35	MOL002894	Berberrubine	35.17	0.73	HB
36	MOL005438	Campesterol	37.58	0.71	HB
37	MOL006392	Dihydroniloticin	36.43	0.82	HB
38	MOL006401	Melian one	40.53	0.78	HB
39	MOL006413	Phellochin	35.41	0.82	HB
40	MOL006422	Thalifendine	44.41	0.73	HB
41	MOL013352	Obacunone	43.29	0.77	HB
42	MOL000088	*β*-Sitosterol 3-O-glucoside_qt	36.91	0.75	CZ
43	MOL000092	Daucosterin_qt	36.91	0.76	CZ
44	MOL000094	Daucosterol_qt	36.91	0.76	CZ
45	MOL000179	2-Hydroxyisoxypropyl-3-hydroxy-7-isopentene-2,3-dihydrobenzofuran-5-carboxylic	45.2	0.2	CZ
46	MOL000184	NSC63551	39.25	0.76	CZ
47	MOL000186	Stigmasterol 3-O-*β*-D-glucopyranoside_qt	43.83	0.76	CZ
48	MOL000188	3*β*-Acetoxyatractylone	40.57	0.22	CZ
49	MOL000085	*β*-Daucosterol_qt	36.91	0.75	CZ, NX
50	MOL000173	Wogonin	30.68	0.23	CZ, NX
51	MOL000098	Quercetin	46.43	0.28	NX, HB
52	MOL00358	*β*-Sitosterol	36.91	0.75	NX,HB
53	MOL000449	Stigmasterol	43.83	0.76	NX, HB
54	MOL000785	Palmatine	64.6	0.65	NX, HB
55	MOL001454	Berberine	36.86	0.78	NX, HB
56	MOL001458	Coptisine	30.67	0.86	NX, HB
57	MOL002643	Delta-7-stigmastenol	37.42	0.75	NX, HB

## Data Availability

The datasets used and/or analyzed during the current study are available from the corresponding author on reasonable request.

## Abbreviations

SMS: San-Miao-San
OA: Osteoarthritis
TCMSP: Traditional Chinese Medicine Systems Pharmacology
OMIM: Online Mendelian Inheritance in Man
TTD: Therapeutic Target Database
PharmGKB: Pharmacogenetics Knowledge Base
PPI: Protein-protein interaction
DAVID: Database for Annotation, Visualization, and Integrated Discovery
TNF: Tumor necrosis factor
IL: Interleukin
TCM: Traditional Chinese medicine
CZ: Cang Zhu/Rhizoma Atractylodis
HB: Huang Bo/Cortex Phellodendri
NX: Niu Xi/Achyranthis Bidentatae
OB: Oral bioavailability
DL: Drug-likeness
PTGS: Prostaglandin-endoperoxide synthase
AR: Androgen receptor
ADRB2: Beta-2 adrenergic receptor gene
BC: Betweenness centrality
CC: Closeness centrality
DC: Degree centrality
EC: Eigenvector centrality
IC: Information centrality
LAC: Local average connectivity-based method
ERS: Estrogen receptor
FOS: Protooncogene c-Fos
Jun: Transcription factor AP-1
Myc: Myc protooncogene protein
GO: Gene ontology
KEGG: Kyoto Encyclopedia of Genes and Genomes
MAPK: Mitogen-activated protein kinase
BP: Biological processes
CC: Cell composition
MF: Molecular function
PDB: Protein Data Bank
ROS: Reactive oxygen species
ERK: Extracellular regulated protein kinases
NRF2: Nuclear factor erythroid 2-related factor 2
AP-1: Activator protein-1
MMP: Matrix metalloproteinase
AP-1: Activator protein-1
JNK: c-Jun N-terminal kinase
NF: Nuclear factor
NO: Nitric oxide
RANKL: Nuclear factor- (NF-) ĸB ligand
TRAF6: TNF receptor-associated factor 6
TLR: Toll-like receptor
STRING: Search Tool for the Retrieval of Interacting Genes/Proteins
FDR: False discovery rate
2D: Two-dimensional.

## Supplementary Materials

Supplementary Materials The data used to support the findings of this study are available in Supplementary Tables S1–S13.
